# Review of fragmentation of synthetic single‐stranded oligonucleotides by tandem mass spectrometry from 2014 to 2022

**DOI:** 10.1002/rcm.9596

**Published:** 2023-08-02

**Authors:** Fabien Hannauer, Rachelle Black, Andrew D. Ray, Eugen Stulz, G. John Langley, Stephen W. Holman

**Affiliations:** ^1^ Chemistry, Faculty of Engineering and Physical Sciences University of Southampton Southampton UK; ^2^ New Modalities & Parenteral Development, Pharmaceutical Technology & Development, Operations AstraZeneca Macclesfield UK; ^3^ Chemical Development, Pharmaceutical Technology & Development, Operations AstraZeneca Macclesfield UK

## Abstract

The fragmentation of oligonucleotides by mass spectrometry allows for the determination of their sequences. It is necessary to understand how oligonucleotides dissociate in the gas phase, which allows interpretation of data to obtain sequence information. Since 2014, a range of fragmentation mechanisms, including a novel internal rearrangement, have been proposed using different ion dissociation techniques. The recent publications have focused on the fragmentation of modified oligonucleotides such as locked nucleic acids, modified nucleobases (methylated, spacer, nebularine and aminopurine) and modification to the carbon 2′‐position on the sugar ring; these modified oligonucleotides are of great interest as therapeutics. Comparisons of different dissociation techniques have been reported, including novel approaches such as plasma electron detachment dissociation and radical transfer dissociation. This review covers the period 2014–2022 and details the new knowledge gained with respect to oligonucleotide dissociation using tandem mass spectrometry (without priori sample digestion) during that time, with a specific focus on synthetic single‐stranded oligonucleotides.

## 1 INTRODUCTION

**FIGURE 1:**
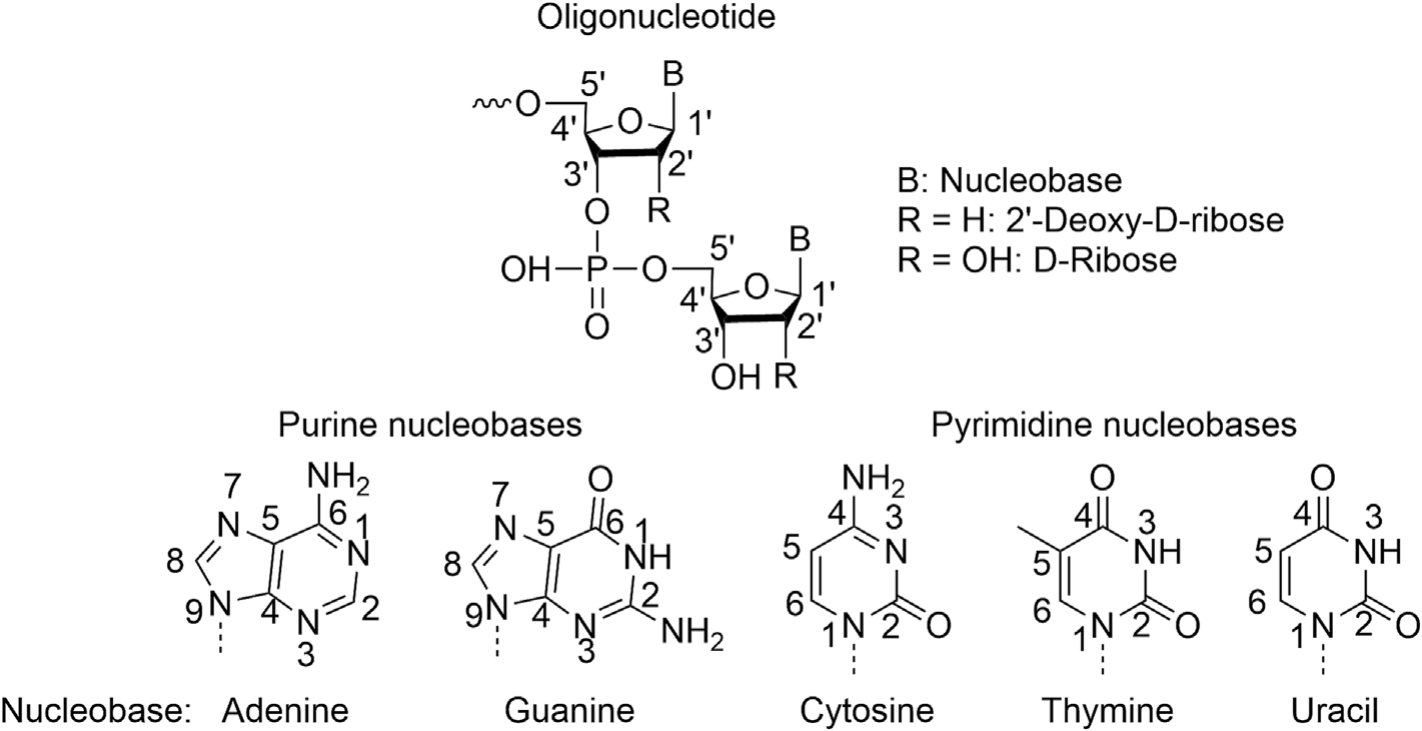
Structures of nucleobases and oligonucleotide. Reproduced from Hannauer et al.^10^

An oligonucleotide, such as DNA or RNA, is a linear polymer of sugars, nucleobases and phosphate groups and was first identified in biological systems. Oligonucleotides are of great interest in the pharmaceutical industry as therapeutics and have different mechanisms in the body, which are dependent upon the interaction of the drug and its target. Novel mechanisms of action are possible, which have been used to develop drugs for ‘undruggable’ diseases or those with limited treatments, for example, Duchenne muscular dystrophy or spinal muscular atrophy, as well as for cancer or in the use of vaccines for Covid‐19.^1^, ^2^, ^3^, ^4^, ^5^, ^6^, ^7^, ^8^, ^9^ The smallest part of an oligonucleotide is a nucleoside, which is composed of a sugar (five carbon atoms with an oxygen atom in a cycle) and one of five nucleobases attached to the carbon 1′ of the sugar (Figure 1). The five nucleobases are adenine (A), guanine (G), cytosine (C) and thymine (T) for DNA sequences or A, G, C and uracil (U) for RNA sequences. The other difference between DNA and RNA sequences is the chemical group bonded to the carbon 2′, which is OH for RNA and H for DNA. When a phosphate backbone, called a linker, is added to the carbon 5′ or 3′, a nucleotide is obtained. Finally, to form an oligonucleotide, which is the succession of a few nucleotides, one sugar is connected from its carbon 5′ to the carbon 3′ of the next sugar via a linker.

**FIGURE 2:**
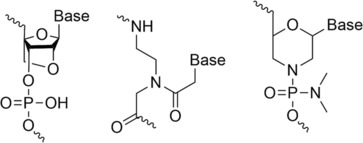
From left to right: structures of LNA, PNA and morpholino phosphoroamidate.

The first oligonucleotide drug approved by the US Food and Drug Administration was fomivirsen in 1998 with the linker modified as phosphorothioate (PS).^1^ Since then, several oligonucleotides have been approved with different modifications to improve their delivery and affinity to the target and their nuclease resistance.^6^, ^11^, ^12^ The main modifications are located on the carbon 2′ as well as to the nucleobase, linker and the terminals of the sequence. Those modifications are classified in different generations that correspond to the order of when they were first synthesised/discovered. The first generation of oligonucleotides corresponded to the modification of phosphodiester backbones such as PS, methyl phosphonate or phosphoroamidate, which increased resistance to nuclease digestion and produced the desired RNase H activity but exhibited undesirable side‐effects and inflammatory responses.^6^, ^12^, ^13^, ^14^, ^15^ Second‐generation oligonucleotides were developed to overcome this deficiency, with the 2′‐hydroxyl group of the ribose modified, limiting proinflammatory effects, proving less toxic and demonstrating greater specificity.^6^, ^13^, ^14^ Improved binding ability as well as nuclease resistance was also achieved.^12^, ^15^, ^16^ This modification includes methoxy and methoxyethyl substituents. Finally, the third generation corresponds to the modification of the furanose ring along with modifications of the phosphate linkage or ribose, as well as of nucleotides. This generation of oligonucleotide has even greater binding affinity to RNA and DNA to modulate gene expression or induce mutation, as well as more desirable pharmacokinetic profiles and stability to enzymatic degradation.^6^, ^12^, ^13^, ^14^, ^16^ These include locked nucleic acids (LNA), peptide nucleic acids (PNA) and morpholino phosphoroamidates (Figure 2).

One of the commonly used techniques to analyse synthetic oligonucleotides is mass spectrometry (MS), with the first report of its application in 1982 by Grotjahn et al. using fast atom bombardment MS.^17^ Since then, different types of oligonucleotides have been characterised thanks to the emergence and widespread use of soft ionisation techniques, most notably matrix‐assisted laser desorption/ionisation (MALDI) and electrospray ionisation (ESI).^18^, ^19^, ^20^, ^21^

Oligonucleotides can be analysed by MS with and without prior digestion.^22^, ^23^, ^24^ When digestion is used, the oligonucleotide analysed is cut at a specific part (either enzymatically or chemically) of its sequence, and the mix of fragments is analysed using MS. This process is called bottom‐up analysis as the determination of the sequence is achieved by the superposition of different digested fragments. When intact oligonucleotides are analysed by tandem MS, also called MS/MS, the process is called top‐down analysis. The molecular weight of the initial sequence is first detected, followed by activation of the precursor ion(s) to generate product ions, with which structural information can be deduced. By using a digestion method, it is not possible to determine the entire sequence in a single step, unless different digestions are performed in parallel, which are site‐specific or non‐specific.^25^, ^26^ The use of several digestions with complementary enzymes of different cleavage specificity generates a range of fragments with overlapping sequences, which allows the overall nucleotide sequence to be deduced. However, those overlapping sequences can have multiple matches making the data complicated, uncertain and ambiguous to attribute.^26^, ^27^ Furthermore, sample preparation can be time consuming due to the need to add a denaturant, for example, 3‐hydroxypicolinic acid, and allow enough time for sufficient digestion to obtain full characterisation.^22^ Furthermore, different digestion methods exist, and each will not give the same fragments.^27^, ^28^, ^29^ Therefore, it is important to select the most appropriate approach, as dictated by the oligonucleotide sequences and modifications under consideration. Non‐specific digestion can be used for oligonucleotides that are resistant to enzymatic digestion (and thus prevent analytically useful data being obtained) but provides a greater data interpretation challenge due to the lack of directionality of cleavage as any potential sites can be cleaved.^28^ The other disadvantage of digestion methods is the high amount of sample used and the time added to the workflow due to the need for several processing steps, but also the optimisation of the method to obtain sufficient digestion to acquire full sequence coverage in the downstream MS analysis.^26^, ^28^, ^29^ Improvements in digestion efficiency have been developed, such as a single‐step sample preparation method, which enables greater throughput,^30^ though these improvements need to be evaluated using different sequences and modifications to show any limitations.

The two main ionisation techniques employed are MALDI and ESI. Whilst MALDI is often used for oligonucleotide analysis, the need for chromatographic separation in many cases makes ESI the most commonly used ionisation technique due to easier hyphenation with liquid chromatography.^14^ Negative ion ESI is typically used as greater sensitivity is achieved by virtue of oligonucleotides readily deprotonating along the highly acidic phosphate backbone.^28^ Furthermore, by using ESI, multiple charge states are obtained allowing analysis using mass analysers with more limited *m*/z ranges. By contrast, MALDI typically generates singly charged molecules, thus requiring a time‐of‐flight mass analyser with its wider *m*/*z* range to detect the ionised species. Whilst analysis of very large oligonucleotides with >450 residues has been reported,^19^ the approach is not routinely used due to challenges associated with analyte detectability and spectral complexity.^22^

**FIGURE 3:**
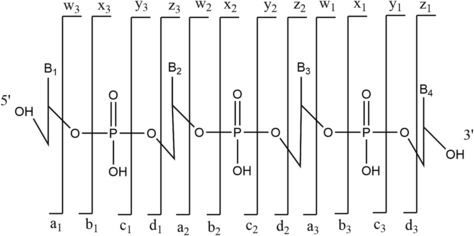
McLuckey nomenclature for oligonucleotide fragmentation.

Tandem MS is an important technique to deduce the sequence of an oligonucleotide. Here, a precursor ion with a specific *m*/*z* is isolated and subsequently fragmented to obtain a product ion spectrum. The most used ion activation technique here is collision‐induced dissociation (CID). Complementary fragmentation information can be delivered using other ion activation techniques such as infrared multiphoton dissociation (IRMPD),^31^, ^32^, ^33^ electron photodetachment dissociation (EPD),^34^ electron capture dissociation (ECD),^35^ electron transfer dissociation (ETD),^36^, ^37^ electron transfer/collisionally activated dissociation (ETcaD),^37^ negative electron transfer CID (NET‐CID),^38^, ^39^ blackbody infrared radiative dissociation (BIRD)^40^ and electron detachment dissociation (EDD).^41^ Furthermore, ion mobility spectrometry (IMS) can be used to complement tandem MS techniques and yield greater understanding of oligonucleotide fragmentation. IMS allows for the separation of different length oligonucleotides, identification of w and y product ions (Figure 3) depending on their ion mobility,^25^ separation of the different charge states,^42^ separation of nucleotides and nucleosides,^43^ separation of folded and unfolded oligonucleotides and separation of isobaric ions, depending on their conformation and the ion mobility resolution.^44^

For tandem MS or in‐source CID (isCID), fragmentation is typically observed around the phosphate backbone. McLuckey introduced a general nomenclature for those ions, which is based on the nomenclature used for the fragmentation of peptide ions^45^ (Figure 3). It has been adopted by the MS community as the standard approach to annotate oligonucleotide MS/MS data 

**FIGURE 4:**
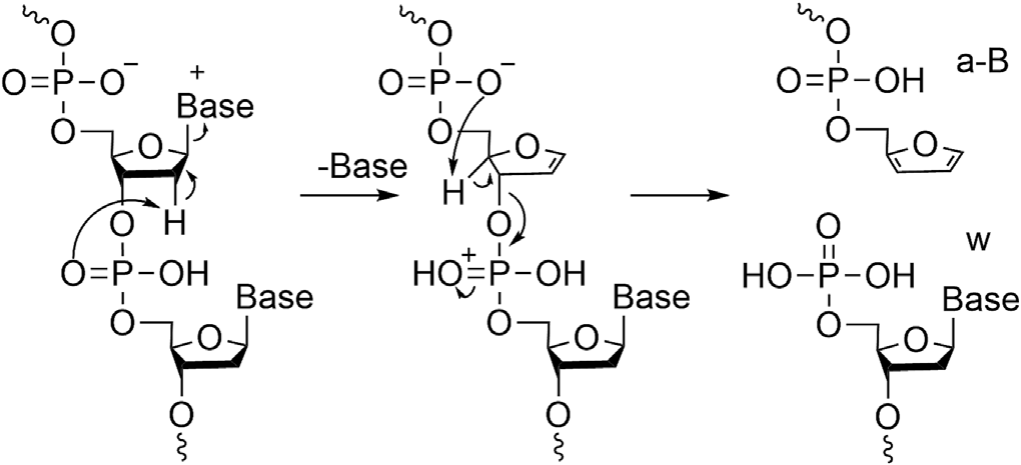
Fragmentation pathway for the formation of a‐B and w in DNA sequences. Adapted from Wan et al.^50^

The most commonly used ion activation technique for the analysis of oligonucleotides has been CID. CID can be performed (broadly) in one of two ways. On‐resonance CID involves exciting a specific precursor ion using a voltage that is at the same frequency as the oscillation of an ion inside an ion trap. This leads to selective excitation, such that when product ions are formed, they do not dissociate further (as their oscillation frequency differs and is not in resonance with the applied RF voltage). Conversely, in off‐resonance CID (inside a collision cell), there is no selectivity as to which ions are excited, and consequently, newly formed product ions can decompose further. Higher‐energy collision dissociation (HCD) was commercialised by Thermo Scientific but operates in the same manner as off‐resonance CID and is thus essentially a synonym. The first publications of CID (both off‐ and on‐resonance) of oligonucleotide ions (both DNA and RNA)^39^, ^46^, ^47^, ^48^, ^49^, ^50^, ^51^, ^52^, ^53^ demonstrated that, in general, a‐B and w ions are obtained for DNA and c and y ions for RNA with reduced loss of base due to the presence of 2′‐OH, which stabilises the *N*‐glycosidic bond on the nucleobase.^54^ The formation of a‐B and w ions can follow several different pathways, as summarised by Wu and McLuckey^55^ and Monn and Schürch.^48^ One example is via a zwitterionic intermediate followed by cleavage at the 3′ C–O bond (Figure 4).^48^, ^49^, ^55^

**FIGURE 5:**
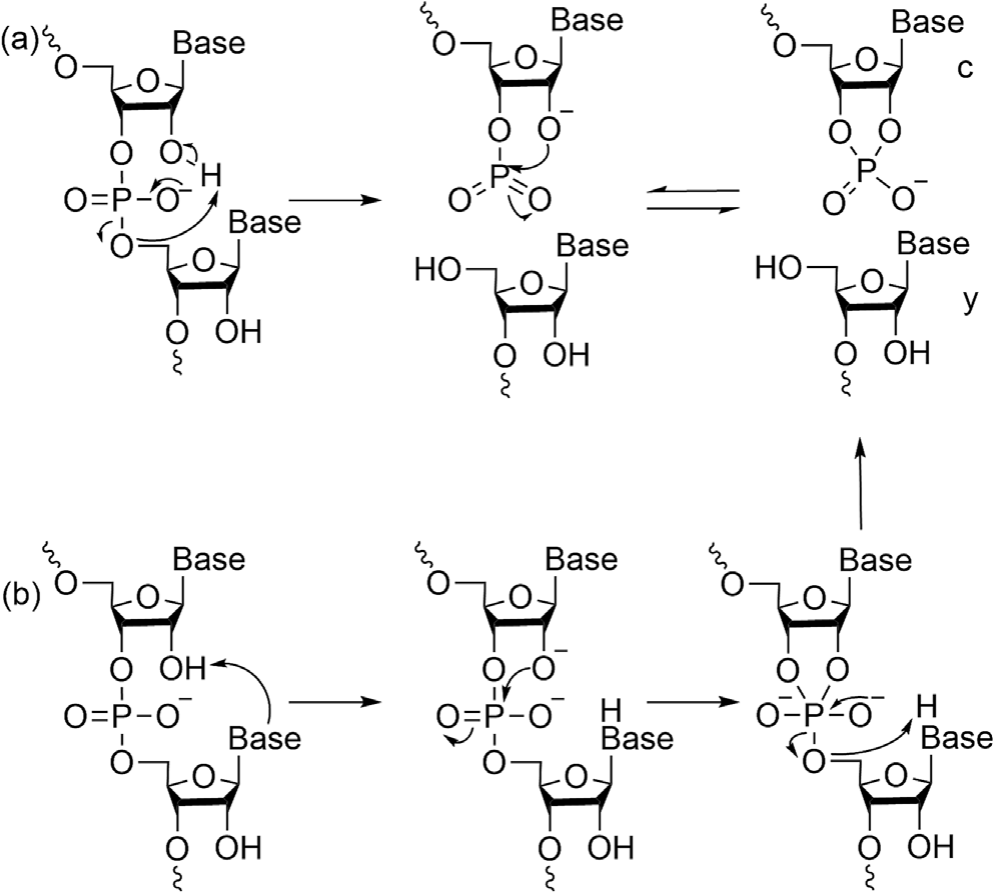
Dissociation pathway for the formation of c and y in RNA sequence with (A, B) two different mechanisms. Adapted from Tromp and Schürch.^54^

Conversely, c and y ions in RNA are formed via an intermolecular cyclic transition state between the 2′‐hydroxyl hydrogen atom and the oxygen of 5′‐phosphate, with two mechanisms proposed (Figure 5).^51^, ^54^ It is not uncommon for RNA and DNA to form the same product ions via different dissociation pathways using CID, which have been summarised by Andersen et al. ^47^ and by Monn and Schürch.^48^ When oligonucleotides are modified on the 2′‐position, different types of product ions are obtained with no preferential dissociation pathways observed; cf. a‐B and w ions for DNA.^56^, ^57^ However, when a mixture of 2′‐modified and unmodified oligonucleotides was analysed, a preferred mechanism was observed, and the gas‐phase stability of the 3′‐side backbone linkage follows the order: 2′‐F > 2′‐OMe > 2′‐H > 2′‐OH.^56^ Pourshahian has reviewed the differences observed between different types of fragmentation used such as IRMPD, UV, EPD, ECD, EDD, ETcaD, NET‐CID and ETD.^28^

The characterisation of oligonucleotides is an important step to determine their sequence. To understand how oligonucleotide ions fragment, different mechanisms have been proposed for DNA and RNA and some reviews have been published.^14^, ^28^, ^46^ Two of the main reviews on mechanisms of dissociation of oligonucleotides have been written by Schurch^14^ and Pourshahian^28^ but are both limited up to 2013. Since then, some reviews have been published but they are generalised on how oligonucleotides can be synthetised, degraded, and analysed rather than on their gas‐phase ion chemistry.^58^, ^59^, ^60^, ^61^, ^62^, ^63^ This review covers the literature published from 2014 to 2022 on the dissociation of synthetic single‐stranded oligonucleotide by tandem MS without digestion. Furthermore, the use of IMS can help to understand the mechanisms of oligonucleotide fragmentation as discussed below.

## 2 MECHANISM OF FRAGMENTATION FOR DNA OR RNA

**FIGURE 6:**
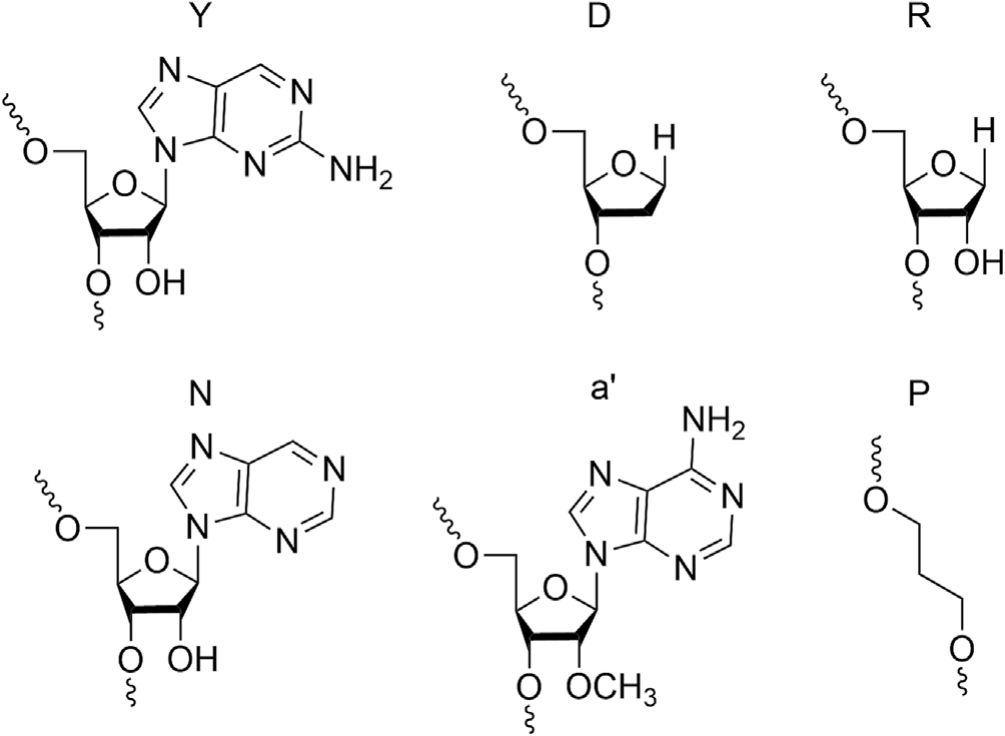
Structures of different modified nucleosides. Y: 2‐aminopurine; D: 2′‐deoxyribose spacer; R: ribose spacer; N: nebularine; a′: 2′‐OCHE adenosine; P: propanediol linker. Analysed in Riml et al.^64^

**FIGURE 7:**
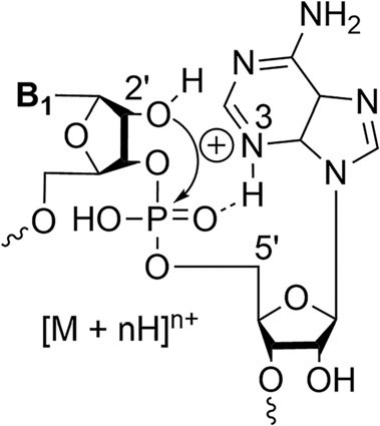
Proposed mechanism for phosphodiester backbone bond cleavage for [M + *n*H]^*n*+^ ions of RNA in CID (off‐resonance). Reproduced from Fuchs et al.^65^

Riml et al.^64^ studied the mechanism of dissociation for modified RNA to understand the phosphodiester bond cleavage by CID (off‐resonance) in positive and negative ESI. They observed that the base loss is more abundant for product ions with 5′ terminus, such as a and c ions, and increases with increasing energy except for a ions, which stay at the same extent of the base loss. They suggest that the base loss plays a critical role only for the formation of a and w ions but not in phosphodiester bond cleavage into c and y ions. Finally, they suggest that backbone cleavage into a and w product ions involves a negative charge compared to c and y product ions, which can proceed in the presence of both positive and negative charges with the facilitation of backbone cleavage into c and y product ions with net positive charges. They observed that more backbone cleavage is obtained into c and y product ions on the 5′‐side of A residues in CID (off‐resonance) for [M + *n*H]^*n*+^ but not for [M − *n*H]^*n*−^ ions. They suggest this observation is due to an intramolecular charge distribution according to simple coulombic repulsion in largely extended positive and negative ion structures immediately before dissociation. Moreover, the effect of A on backbone cleavage decreases significantly when the collision energy is increased, which suggests that this dissociation is based on structural elements that are not preserved at elevated internal energies. The same observation is obtained for deprotonated G but with a dependence on the RNA sequence. Both G and A show no correlation with the location of charge immediately before backbone cleavage. To validate those suggestions, they analysed different modified oligonucleotides (Figure 6). When 2‐aminopurine (Y) or nebularine (N) is present in the sequence, no significant effect on backbone cleavage is obtained compared to A. They observed that when the 2′‐OH is replaced 2′‐deoxyribose spacer (D), 2′‐OCHE adenosine (a′) or propanediol linker (P), the backbone cleavage into protonated c and y ions is inhibited. However, when the modification ribose spacer (R) is present, increased yields are obtained for protonated c and y product ions. They suggest that the nucleobase on the 3′‐side of the phosphodiester bond may hinder the nucleophilic attack of the ribose 2′‐OH on the phosphorus by restricting the conformational flexibility of the ribose. Using negative ESI, they obtained a similar spectrum with the modification a′, D or P without signals arising from c and y ions. With R, they did not see an evident effect of the modification and suggest that the higher collision energy applied in negative ion mode, the greater the restoration of ribose conformational flexibility. They suggest that intramolecular proton transfer in vibrationally excited [M + *n*H]^*n*+^ ions can also involve the phosphodiester moiety. On the other hand, proton transfer in [M − *n*H]^*n*−^ ions can proceed via both the phosphodiester groups and the deprotonated nucleobases. Their observations show that the ribose 2′‐OH group is involved in RNA backbone cleavage into c and y product ions by CID (off‐resonance) for both positive and negative ions. Furthermore, they suggest that the phosphodiester bond cleavage does not directly involve charged sites. They propose that the dissociation into complementary c and y ions is not a simple bond cleavage but proceeds via a more complex rearrangement reaction with an intermediate such as a cyclic pentacoordinate oxyphosphorane for positive and negative ions. They propose a stepwise mechanism similar to the transesterification and cleavage steps involved in RNA hydrolysis in solution (Figure 7). First, a pentacoordinate oxyphosphorane intermediate is formed by a nucleophilic attack of a ribose 2′‐OH group on the adjacent phosphorus. They propose that nucleophilic attack of the 2′‐OH group on the phosphorus can be facilitated by hydrogen bonding between a non‐bridging oxygen atom of the phosphoric acid diester group and A protonated at N3, which is the preferentially protonated site in the gas phase. When the internal energy of the ion is further increased, the RNA chain becomes more extended, due to the breaking of the hydrogen bonds, and protons can redistribute accordingly to simple Coulombic repulsion, after which even more added energy finally gives rise to the phosphodiester backbone cleavage into c and y product ions. The authors propose a similar situation for negative ions where a neutral instead of a protonated base forms a hydrogen bond with a non‐bridging oxygen of the phosphodiester group to facilitate the nucleophilic attack. Finally, they defined an energy barrier for each step, which can be ranked as nucleophilic attack to form intermediate < breaking of hydrogen bonds and extension of RNA structure < intramolecular proton transfer < phosphodiester bond cleavage into c and y fragments where the energy barrier involved in the dissociation of negatively charged RNA is higher than for positively charged RNA due to the need to apply higher energies for [M − *n*H]^*n*−^ ions with CID (off‐resonance).

A few years later, the same research group published other observations, using again positive and negative ESI, which support the previous mechanism proposed.^65^ The authors removed the basic N3 site of A, which abrogated preferential phosphodiester backbone bond cleavage. They also removed the N1 or N7 but did not observe any interference with the phosphodiester backbone bond cleavage. They conclude that multiply protonated RNA under CID (off‐resonance) forms ionic hydrogen bonds between the N3 protonated A and an oxygen of the adjacent phosphodiester backbone moiety on its 5′‐side, which facilitates nucleophilic attack of the 2′‐oxygen onto the phosphorus on its 5′‐side and dissociation into c and y product ions. Furthermore, they observed that the phosphodiester backbone bond cleavage next to A in the gas phase is affected by the pH of the solution. Lowering the pH reduced the yield of c and y dissociation for the A located at the terminals compared to the one towards the middle of sequence for which increased c and y ion formation was observed. Therefore, the authors proposed that a part of the protonation in solution is transferred into the gas phase in ESI as proton affinity and Coulombic repulsion oppose the observed effect of decreasing pH.

**FIGURE 8:**
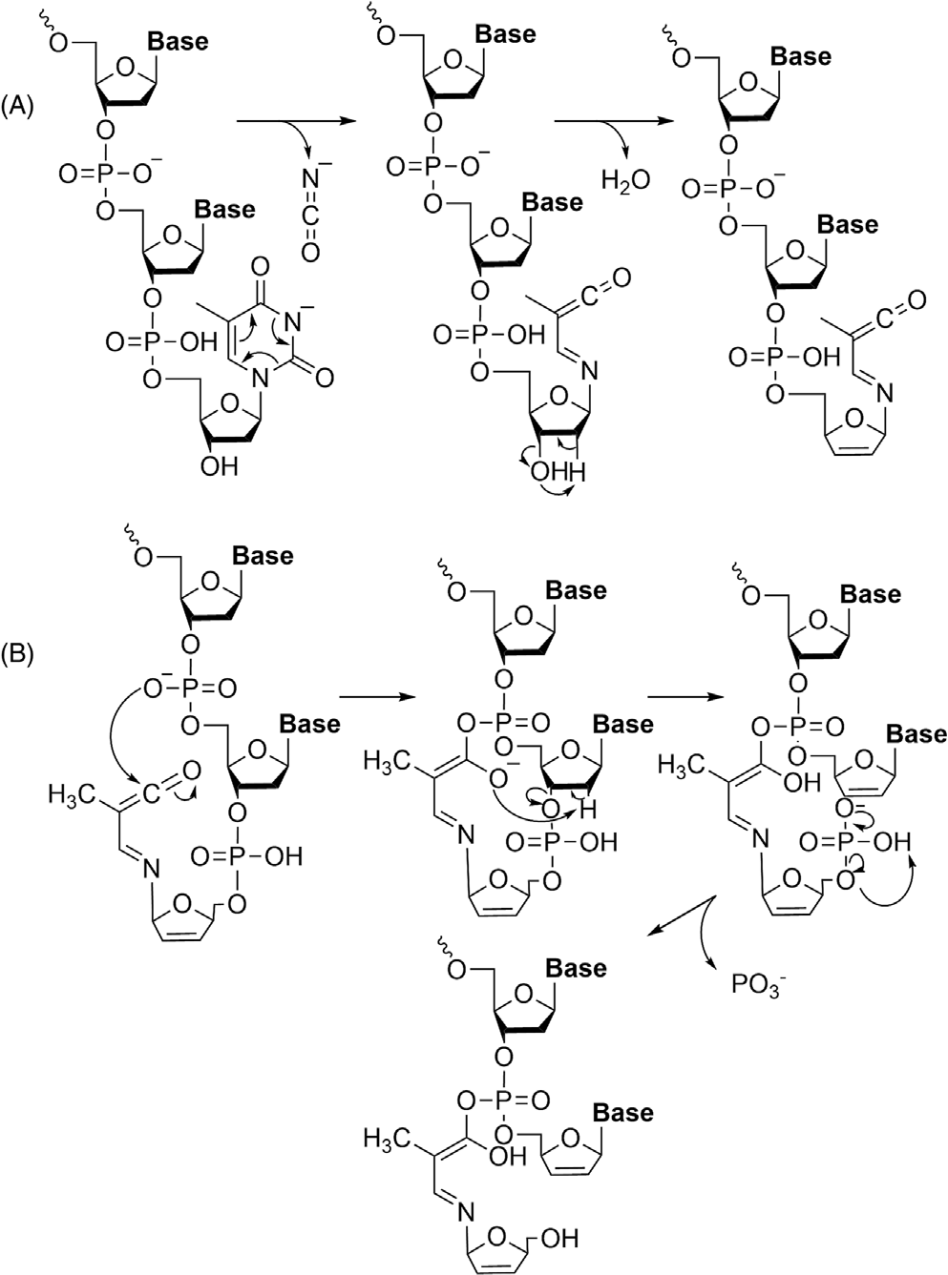
(A) Proposed mechanism for the release of NCO^−^ upon CID (on‐resonance) of highly negatively charged oligonucleotide. (B) Postulated mechanism for metaphosphate excision as subsequent reaction to NCO^−^ and water loss. Reproduced from Nyakas et al.^66^

Nyaka et al. reported a new dissociation pathway for DNA as well as for RNA, using negative ESI, which involves the loss of NCO^−^.^66^ The authors observed this dissociation by CID (on‐resonance) only for highly charged oligonucleotides when pyrimidine is present at the terminal 3′ or 5′ as the source of cyanate loss. Moreover, the loss of NCO^−^ is more observable when the oligonucleotide has a terminal thymine instead of a cytosine or a pyrimidine base that is located at the 3′‐end instead of the 5′‐end. They could not observe any loss of NCO^−^ from an internal pyrimidine. Furthermore, when the sequence length increases, NCO^−^ loss decreases, and as a consequence, it can be only observed for short oligonucleotides up to 12‐mers. They observed that first NCO^−^ is lost, followed by backbone dissociation to obtain [w‐NCO^−^]^−^ or [a‐B‐NCO^−^]^−^. They also observed [M − NCO^−^ − PO_3_^−^ − H_2_O]^3−^. They proposed three different dissociation mechanisms, ruling out two due to inconsistencies with experimental observations. The most likely mechanism for the release of NCO^−^ by CID (on‐resonance) of highly negatively charged oligonucleotides using the thymine model base is shown in Figure 8A. The authors also observed that the terminal hydroxyl group was not involved in the generation of the cyanate anion and corresponded to the only source of water loss.^66^ The loss of water was not observed when the OH terminal was absent. By analysing modified phosphate with no charge at the different positions, they observed that a phosphate group is not a crucial prerequisite for the NCO^−^ loss dissociation pathway as they still observed a low abundance peak for the cyanate release. Hence, they also rejected the hypothesis that the terminal phosphate oxygen is exclusively responsible for the initiation of cyanate release. Based on this observation, they proposed the mechanism in Figure 8A, which constitutes the main pathway for the release of cyanate. In this mechanism, the loss of NCO^−^ is initiated by initial deprotonation of the thymine at N3, which means that this mechanism is independent of any other component of the oligonucleotide. They suggest that ‘a negative charge in a fully deprotonated oligonucleotide may reside on a nucleobase for a certain period of time’ as T is the most acidic base in DNA. They support this mechanism by observing [M − NCO]^−^ peak from in‐source decay, which supports the theory of the initial deprotonation of the terminal thymine, even during the ionisation process, that releases the cyanate by a retro‐Diels–Alder mechanism, as shown in Figure 8A, if the Coulombic repulsion of the oligonucleotide is large enough. In the case of RNA, they observe that a sequence with a 3′‐terminal uracil gives a more pronounced [M − NCO]^−^ signal than for a 5′‐terminal U or a terminal C. Moreover, a small loss of water (less than 1% relative intensity) is observed, which they suggest is due to the 2′‐hydroxyl group interfering with the elimination of H_2_O. They suggest that the 2′‐proton is involved in the process of water loss rather than the 4′‐proton. Finally, the loss of cyanate is exclusively observed from the 3′‐terminal uracil and not from an internal position. Moreover, they observed that the loss of PO_3_^−^ in RNA occurs between the two last bases when NCO^−^ and water are released. This loss of PO_3_^−^ is more observed at higher charge, indicating that it is dependent on the charge state and the length of the sequence.

By detailed analysis of a number of different oligonucleotide sequences, the authors proposed a mechanism for the release of the terminal PO_3_^−^ from the [M − NCO]^−^ precursor ion (Figure 8B). In this mechanism, the deprotonated oxygen from the phosphate group situated before the two last bases attacks the ketene structure, generating a covalent C–O bond. Then, the 2′‐proton is abstracted by the oxygen from the ketene to initiate 3′‐C–O bond cleavage, but not the 4′‐proton, similar to the pathway for water loss. As for the abstraction of H_2_O, the loss of PO_3_^−^ is exclusively observed for highly charged DNA sequences (for ions with >60% deprotonation relative to their theoretical maxima based on the reported data), whereas RNA underwent a negligible amount of PO_3_^−^ loss. They suggest that the 2′‐position is involved in the process of PO_3_^−^ loss.

**FIGURE 9:**
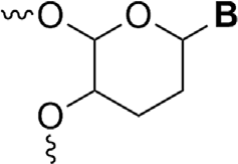
Structure of homo‐DNA.

The authors support those mechanisms with the analysis of homo‐DNA that exhibits a hexose sugar unit instead of a pentose (Figure 9). According to those two mechanisms, homo‐DNA could undergo loss of NCO^−^, but they observed that the loss of PO_3_^−^ and water is impeded by the hexose sugar unit, as the abstraction of the 2′‐proton induces neither water loss nor cleavage of the 4′‐C–O bond (3′‐C–O for DNA). They observed that the loss of NCO^−^ is more prevalent for homo‐DNA than for unmodified DNA. As a consequence, they suggest that the 2′‐position is very likely to be involved in the process of water loss and PO_3_^−^ excision.

## 3 CHARACTERISATION OF THIRD‐GENERATION OLIGONUCLEOTIDES

The most recent generation of oligonucleotides, so‐called ‘third generation’, contain modifications along the furanose ring, phosphate linker, ribose or nucleotides to improve their stability and have better target affinity.

### 3.1 Characterisation of methyl nucleoside isomers

**FIGURE 10:**
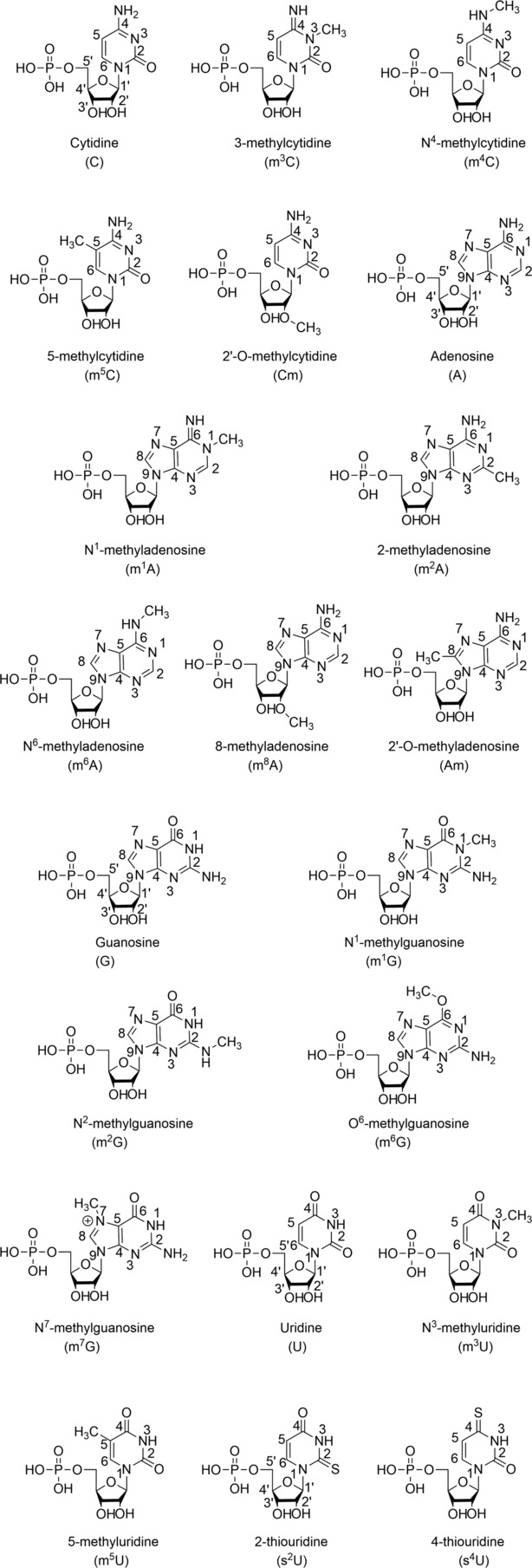
Structures of the methylcytidine, methyladenosine, methylguanosine, methyluridine and thiouridine monophosphate isomers analysed in Jora et al.^67^ and Li et al.^68^

To characterise the modification on the nucleobase, Jora et al.^67^ used MS^*n*^ to first isolate the precursor oligonucleotide ion followed by dissociation where each observable nucleobase ion can then be isolated and dissociated. The authors used this method with HCD (off‐resonance) or CID (on‐resonance) with an Orbitrap, in positive ESI mode, to differentiate positional isomers such as C, 3‐methylcytidine (m^3^C), N^4^‐methylcytidine (m^4^C), 5‐methylcytidine (m^5^C), A, N^1^‐methyladenosine (m^1^A), 2‐methyladenosine (m^2^A), N^6^‐methyladenosine (m^6^A), 8‐methyladenosine (m^8^A), G, N^1^‐methylguanosine (m^1^G), N^2^‐methylguanosine (m^2^G), N^7^‐methylguanosine (m^7^G), U, N^3^‐methyluridine (m^3^U), 5‐methyluridine (m^5^U), 2‐thiouridine (s^2^U) and 4‐thiouridine (s^4^U) (Figure 10). It has been shown that even when positional isomers co‐elute, it is possible to deconvolute the individual ‘fingerprints’ from the mixture data to identify which isomers are present. To achieve this, the authors first fragmented the different nucleosides, but they did not observe any specific pathway from the different isomers. They decided to directly fragment the nucleobase from the different nucleosides and proposed that product ions with relative ion abundances of ≥15% should be used for developing an HCD (off‐resonance)‐based nucleoside product ion fingerprinting approach. Finally, the presence of the modification can be confirmed, but to determine its exact position, further MS^*n*^ of different product ions is needed or the use of IMS.

**FIGURE 11:**
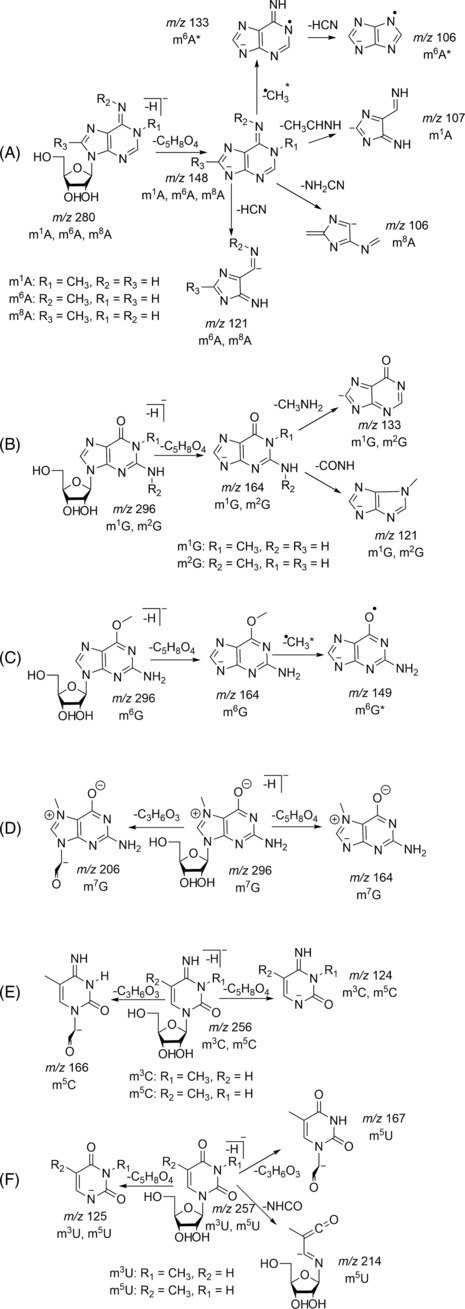
Proposed CID (on‐resonance) pathways for (A) methyladenosine, (B–D) methylguanosine, (E) methylcytidine and (F) methyluridine adapted from Li et al.^68^ Original assignment of loss of NH replaced with re‐interpretation as loss of ^•^CH_3_ as denoted by *, with consequent new proposals of product ion structures.

Two years later, another study of the methyl nucleoside isomers using negative ESI was conducted by Li et al.^68^ The authors compared the various dissociation patterns by using MS/MS or MS^3^ of the nucleosides down to *m*/*z* 100. To do that, they studied the difference between m^1^A, m^6^A, m^8^A, m^1^G, m^2^G, O^6^‐methylguanosine (m^6^G), m^7^G, m^3^C, m^5^C, m^3^U and m^5^U (Figure 10). They could observe some differences between the isomers by only fragmenting the nucleoside. Each different nucleoside gave a specific dissociation pattern, as shown in Table 1. By example, the authors state that ‘the base peak of m^1^A in the MS^3^ dissociation spectrum was *m*/*z* 107 (corresponding to the loss of CH_3_CN), whereas m^6^A was *m*/*z* 133 (corresponding to the loss of NH) and m^8^A was *m*/*z* 121 (corresponding to the loss of HCN)’. The loss of NH is extremely unlikely and would more likely correspond to the loss of a methyl radical. However, the work was conducted using an ion trap mass spectrometer, and no accurate mass data from other approaches were reported. Therefore, the identity of the 15 *m*/*z* unit loss is yet to be conclusively proven. A hypothesised CID (on‐resonance) pathway for the different methyladenosines has been proposed by the authors but without any mechanism to explain it. They repeated the same process for C, U and G and proposed for each a hypothesised CID (on‐resonance) pathway summarised in Figure 11. For C and U, they can be easily distinguished using only MS^2^ by fragmenting the nucleoside. Methylcytidine (*m*/*z* 256) gives a proposed characteristic C_3_H_6_O_3_ loss for m^5^C, which is not observed for m^3^C. A common product ion for these two nucleosides is detected at *m*/*z* 124 (proposed loss of C_5_H_8_O_4_). For methyluridine nucleosides, two specific product ions at *m*/*z* 167 (proposed loss of C_3_H_6_O_3_) and *m*/*z* 214 (proposed loss of NHCO) are detected for m^5^U, which are not observed for m^3^U. Both m^3^U and m^5^U fragment to give a product ion at *m*/*z* 215 via the proposed loss of C_5_H_8_O_4_. Finally, for methylguanosines, only m^7^G can be characterised directly without further dissociation of the nucleobase. Dissociation of the m^7^G precursor ion (*m*/*z* 296) gives two characteristic product ions at *m*/*z* 206 and 164 (proposed losses of C_3_H_6_O_3_ and C_5_H_8_O_4_ respectively), which are not obtained for m^6^G, m^1^G and m^2^G. For those molecules, it is necessary to fragment their nucleobase, for example, dissociation of the m^6^G nucleobase ion at *m*/*z* 164 results in a specific fragment ion at *m*/*z* 149 (proposed loss of NH by the original authors; new proposal here of loss of a methyl radical). The identification of m^1^G and m^2^G depends on the ratio of the peaks obtained at *m*/*z* 133 (proposed loss of CH_3_NH_2_) and *m*/*z* 121 (proposed loss of CH_3_CO by the original authors; new proposal here of loss), after dissociation of those nucleobases. For m^2^G, its base peak is observed at *m*/*z* 121 (proposed loss of CH_3_CO by the original authors; new proposal here of loss of CONH), compared to m^1^G at *m*/*z* 133 (proposed loss of CH_3_NH_2_).

**TABLE 1:** Summary of different dissociation patterns for various nucleosides.

Fragmentation of (at *m*/*z)*	Fragment ion at *m*/*z*	Loss of	Fragmentation of previous fragment ion for	Fragment ion at *m*/*z*	Proposed loss from Li et al.^68^
m^1^A, m^6^A, m^8^A (280)	148	C_5_H_8_O_4_	m^1^A	107	CH_3_CN^a^
			m^6^A	133	NH^a^
			m^8^A	106	NH_2_CN
m^1^G, m^2^G, m^6^G (296)	164	C_5_H_8_O_4_	m^1^G, m^2^G	133	CH_3_NH_2_
				121	CH_3_CO^a^
			m^6^G	149	NH^a^
m^7^G (296)	206	C_3_H_6_O_3_			
	164	C_5_H_8_O_4_			
m^3^C, m^5^C (256)	124	C_5_H_8_O_4_			
m^5^C (256)	166	C_3_H_6_O_3_			
m^3^U, m^5^U (257)	125	C_5_H_8_O_4_			
m^5^U (257)	167	C_3_H_6_O_3_			
	214	NHCO			

^a^ Loss as assigned in the original paper, but extremely unlikely to be correct.

Furthermore, m^5^C, m^4^C, m^3^C, 2′‐*O*‐methylcytidine (Cm), m^8^A, m^6^A, m^1^A and 2′‐*O*‐methyladenosine (Am) have been analysed on cyclic ion mobility (cIM) by Kenderdine et al.^44^ They could easily separate the different nucleosides and identify them by applying 10 passes around the IM device, ejecting some isomers to submit additional passes on the partially resolved ones. They also dissociated them after cIM, which allows the generation of specific product ions, such as loss of methylamine or opening of the pyrimidine ring, depending on the position of the methyl group on the nucleoside.

cIM, CID (on‐resonance) and HCD (off‐resonance) are useful to identify the different positions of the isomer on the nucleobase, but they do not give the exact position of the different modifications in the sequence. To do that, MS^*n*^ is advantageous because it is possible to isolate and dissociate again the product ion to follow the dissociation compared to tandem MS where all the ions from the precursor and product ions can be dissociated again. Then, by overlapping the different results from the different segment of the sequence, it is possible to identify the localisation of the modification in the sequence. However, this may not always be successful, as the depletion of the ion current at each stage of the MS^*n*^ analysis can mean that the limit of detection may be reached before MS data allowing localisation are obtained.

### 3.2 Characterisation of LNA

LNA is one modification included in the third generation of oligonucleotides. This modification is situated on the sugar where the 2′‐O and 4′‐C are joined through a methylene bridge, as shown in Figure 2, which gives a rigid conformation. This rigid conformation mimics that adopted by RNA, which increases the stability of the oligonucleotide sequence and enables enhanced targeting of biological RNA with fewer off‐target effects.^69^, ^70^ A difference of dissociation behaviour, between DNA and LNA for single strands and duplexes by using negative ESI with CID (off‐resonance) and ion mobility, has been published recently by Ickert et al.^71^ The authors observed that at low charge states, such as [M − 4H]^4−^, many more product ions were obtained (174 product ions for a 15‐mer) for LNA compared with that at higher charge states, [M − 9H]^9−^ (45 product ions for the same 15‐mer). The same behaviour is observed for DNA but less significantly (39 product ions for [M − 4H]^4−^ and 22 for [M − 9H]^9−^). This is due to the lack of structural flexibility caused by the additional methylene bridging in LNA, which hinders the base loss, and as a consequence, other dissociation channels occurred with multiple backbone cleavages. In DNA, the main product ions observable are w and a‐B where in LNA the base loss is at very low abundance and no singly negative charged nucleobases were observed. Furthermore, in LNA, different product ions without base loss were obtained with no preference for a specific backbone cleavage position. Those product ions, from a to d and w to z type, were detected at high abundances with a need of lower relative collision energy compared to DNA. As a consequence, a more complex and information‐rich mass spectrum is obtained which is likely to enable better characterisation of the molecules. However, the authors did not assess the extent of internal fragment ion generation in DNA and LNA (see Section 5.1 for the definition of internal fragment ion). It would therefore be interesting to compare the relative propensities of DNA and LNA to generate internal fragments, enabling assessment of the additional information, and potentially increased sequence coverage, that can be obtained compared to product ions from the McLuckey series.

## 4 OTHER MS/MS TECHNIQUES TO CHARACTERISE OLIGONUCLEOTIDES

Oligonucleotides have been characterised mainly by CID (both off‐ and on‐resonance) but also by other techniques such as IRMPD,^31^, ^32^, ^33^ EPD,^34^ ECD,^35^ ETD,^36^, ^37^ ETcaD,^37^ NET‐CID,^38^, ^39^ BIRD^40^ or EDD.^41^ Different observations have been obtained for the different techniques.

Daly et al. analysed different DNA polyanions using EPD.^72^ They concluded that purines readily undergo electron detachment compared to pyrimidines, which is a minor channel and have instead w, a‐B, and base loss product ions. As a consequence a ranking, depending on the nucleobase, can be established as follows for EPD efficiency: G >> A >> T ≈ C.

Furthermore, different activation techniques have been compared to each other to understand the complementarities of the approaches in obtaining higher sequence coverages. Ickert et al.^73^ compared HCD (off‐resonance) to CID (on‐resonance) for short oligonucleotides in negative ESI mode. They observed that CID (on‐resonance) gives mainly abundant [M − *x*H − B]^*x*−^ product ions, which are at low abundance or not detected in HCD (off‐resonance) spectra. HCD (off‐resonance) gives abundant base loss of products such as [c_2_‐A] product ions, which are at very low abundance in CID. In general, HCD (off‐resonance) gives more intense product ions than CID (on‐resonance), but due to their differences, HCD (off‐resonance) and CID (on‐resonance) are orthogonal techniques for oligonucleotide structure elucidation.

Abdullah et al.^74^ also compared HCD (off‐resonance) to CID (on‐resonance), in negative ESI, by analysing different DNA T‐rich sequences, of around 18‐mers, with and without PS modification by applying different normalised collision energies (NCE). When unmodified DNA is analysed, 25% of NCE is necessary to fragment the precursor by CID (on‐resonance) compared to HCD (off‐resonance) where 10% is sufficient to produce product ions. At higher NCE for HCD (off‐resonance), they obtained additional b/x and c/y ions compared to the usual a‐B/w ions in DNA sequences, which are then diminished due to the extensive sequential fragmentation. Those additional product ions are mostly produced from backbone cleavages close to the 5′‐ or 3′‐ends and will only serve to confirm rather than to add any more sequence coverage. By using the optimal collision energy, HCD (off‐resonance) provides higher total product ion intensities than CID (on‐resonance) but with an increase of acquisition time. The authors observed that the loss of base T, such as a‐B(T), was unfavoured for both CID (on‐resonance) and HCD (off‐resonance). However, fewer w ions, complementary to a‐B(T), are present with HCD (off‐resonance) than CID. They observed that HCD (off‐resonance) provides a higher quantity and quality of sequence‐defining fragments compared to CID (on‐resonance) for unmodified DNA. When P=O is replaced by P=S, they observed that an NCE of 25% and above is necessary for CID (on‐resonance) to obtain predominantly a‐B/w‐ions compared to HCD (off‐resonance) where 10% can be used. With the optimum NCE (where the highest fragment intensities and the most diagnostic fragment ions are obtained) for CID (on‐resonance) (35%) and HCD (off‐resonance) (10%), they obtained more intense product ions by HCD (off‐resonance) than CID (on‐resonance). They could not observe any a‐B(T) by CID (on‐resonance) or HCD (off‐resonance), and only one w fragment, complementary to a‐B(T), was observed by HCD (off‐resonance). Furthermore, when P=S is present in the sequence for DNA, other missing a‐B ions are observed compared to unmodified DNA where only a‐B(T) is not observed. However, by applying an NCE of 20% or above for HCD (off‐resonance), they obtained more nucleotide units such as b_n_/x_n_, c_n_/y_n_ and d_n_ ions in the modified DNA compared to the unmodified one, which could give additional sequence‐defining product ions compared to CID. Consequently, HCD (off‐resonance) with multiple NCEs could be used to promote complementary dissociation pathways and a better sequence coverage than CID (on‐resonance) for sequencing T‐rich PS‐modified DNA.

Peters‐Clarke et al. studied negative electron transfer dissociation (NETD) and activated ion NETD (AI‐NETD), for modified RNA and siRNA, and compared them to CID (on‐resonance) and HCD (off‐resonance).^75^ They observed that NETD and AI‐NETD generate mainly non‐complementary d and w product ions for RNA with considerably less base loss and internal product ions. As a consequence, a simpler spectrum is obtained compared to CID (on‐resonance) and HCD (off‐resonance), which have a higher number of peaks corresponding to internal product ions or base loss, which do not add complementary information. When they applied a higher infrared laser power for AI‐NETD, the product ions c, d, w, and y had similar intensity. At even higher power, d and w are diminished, and the spectra look like CID (on‐resonance) or HCD (off‐resonance), and so it is possible, by applying the correct power, to generate the desired product ions. Furthermore, they obtained a good sequence coverage for a 21‐mer with only d and w product ions using NETD and AI‐NETD. To support those results, more variants of oligonucleotides need to be analysed as they only characterised two oligonucleotides, one modified 6‐mer RNA and one 21‐mer siRNA. NETD and AI‐NETD could be complementary techniques to CID (on‐resonance) and IRMPD, or, if utility on a greater number of sequences is demonstrated, they could be used alone for modified and unmodified oligonucleotides.

**FIGURE 12:**
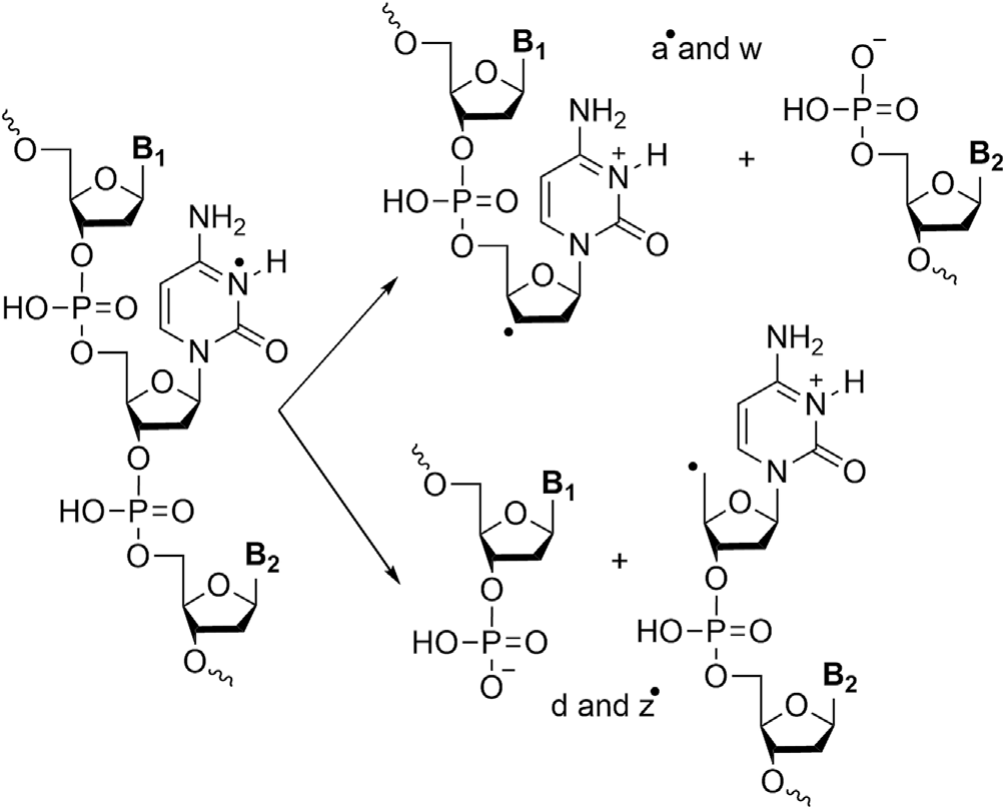
Proposed dissociation products of ETD in positive ion ESI where w or d ion carries a negative charge and the complementary product ion harbours a positive charge. Reproduced from Hari et al.^76^

ETD of oligonucleotides (6‐mers) has also been studied in positive ion ESI by Hari et al.^76^ to produce structural information complementary to that of CID. Initially, after applying electron transfer to triply charged DNA hexamer cations, the authors obtained the radical species [M + 3H + e^−^]^2+•^ and [M + 3H + 2e^−^]^+••^, which are stable. Following supplemental activation using HCD (off‐resonance), backbone cleavage or nucleobase loss was observed, where an order for the reduction of nucleobases is obtained as C > A > G > T. The reduction of the base is followed by cleavage of the C–O bond preferentially at the 5′‐side. Due to the previous order, the reduction of cytosine is preferred, which could be due to favoured electron transfer. Furthermore, cytosine is the favourite to be reduced due to its high proton affinity and could give sequence information complementary to CID (off‐resonance) for C‐rich sequences as well as modified cytosine compared to CID (off‐resonance) where the purines are the preferred cleavage sites. Also, they observed that the cleavage of cytosine next to the terminals is preferred compared to an internal one. They explain this as a charge‐directed process where the protons are pushed to the terminal nucleobases by Coulombic repulsion in electron transfer. When a purine is located at a terminal position, it has a similar probability of being ejected than a cytosine. Moreover, they observed that the loss of nucleobases accounts for only 1–3% of the total signal intensity in the product ion spectrum compared to charge reduction and backbone cleavage. Finally, they did not observe any loss of nucleobases from a backbone fragment such as a‐B, and they suggest that base loss and backbone cleavage are independent processes. For the loss of neutral nucleobases, they proposed the same mechanisms as previously observed,^77^ which is that the unpaired electron on the nucleobase induces the homolytic scission of the *N*‐glycosidic bond. For the dissociation of a^•^/w and d/z^•^ ion pairs in DNA, they propose a pathway with product ion structures, which is obtained after the reduction of the nucleobases by electron transfer (Figure 12). They observed the fragment z_1_^+•^ that proves that the terminal nucleobase retains its proton and proposed that the backbone cleavage is due to electron transfer from the nucleobase onto the phosphate linker rather than by transfer of a hydrogen atom. After this internal electron transfer, they propose that the 5′‐C–O bond is cleaved, resulting in the z^•^ radical ion and a negative charge on the phosphate group, whereas the d ion is a zwitterion with one negative charge on the last phosphate group and two protons on separate nucleobases. They suggest that the internal proton transfer in the d ion may facilitate the separation of the product ions. They still observed the characteristic backbone cleavage as previously observed using ECD,^35^ for a^•^/w and d/z^•^ ion pairs, which is induced by the transfer of H^•^ but as a very minor reaction pathway due to lower ion–electron recombination energies upon electron transfer. For the formation of a^•^/w ion pairs, where no a‐B are observed, the backbone cleavage is independent of nucleobase loss. They suggest that the a^•^/w ion pair is likewise the product of internal electron transfer. a^•^/w ion pairs are the dominant dissociation pathway when cytosine is present at the 5′‐terminal and for pairs of cytosines but d/z^•^ pairs are preferred for internal cytosines. The authors hypothesised that the preference for cleavage of the 5′‐C–O bond rather than the 3′‐C–O bond after the reduction of the cytosine can be due to energetic differences of the σ*‐orbitals involved, which is not fully understood. Consequently, ETD can be a complementary method to CID (off‐resonance) to localise the modifications within the phosphate linker. They obtained the same previous observation for modified sequences such as RNA and methylphosphonate, which means that the mechanism of the backbone cleavage is not affected by structural modifications that preserve the phosphodiester linkage in the backbone. They observed that for 2′,3′‐di‐deoxyglucopyranose, the dissociation yield is increased and homo‐DNA will give more intense backbone product ions compared to unmodified DNA. When DNA and homo‐DNA are present in the same sequence, the backbone cleavage next to the homo‐DNA is preferred. They explain this observation as due to the less effective stabilisation of the radical species in homo‐DNA. Consequently, ETD can give complementary information to CID (off‐resonance) when multiple modifications are present in the sequence as the modifications do not affect the mechanism for ETD. They hypothesise that the high intensity of the DNA radical cations [M + 3H^+^ + 2e^−^]^+••^ and their stability can be due to the stabilisation of the unpaired electron (π–π stacking hypothesis), which prevents the cleavage of the backbone. They support this hypothesis by the observation of increased dissociation yields of the backbone product ions when one nucleobase is replaced by a hydrogen atom in the sequence. They explain this as due to the greater accessibility of the deoxyribose for the electron carrier and the protonation of the oxygen atom in the sugar moiety, which could assist electron transfer. In other words, the DNA radical cation without modification is more stable due to the stabilisation of the unpaired electron by π–π stacking. Furthermore, they suggest that this hypothesis explains the different dissociation yields for the DNA homomers where low‐intensity product ions are obtained for dA6 and dG6 due to the high stacking efficiencies of the purine nucleobases.

Santos et al.^78^ compared HCD (off‐resonance), UVPD using 193 and 213 nm photons and activated EPD (a‐EPD), in negative ESI mode, for top‐down sequencing of two therapeutic oligonucleotides, namely nusinersen and patisiran, without any modification, then with different modifications such as PS, LNA and 2′‐*O*‐methyl and 2‐methoxyethoxy modifications. Nusinersen is an 18‐mer PS 2′‐*O*‐methoxyethoxy antisense oligonucleotide with all cytidines methyl‐modified at the 5‐position. On the other hand, patisiran is a 21‐mer double‐stranded small siRNA. The sense strand contains 2′‐*O*‐methyl in all of the pyrimidine nucleotides (cytidine and uridine), whilst the antisense strand includes 2′‐*O*‐methyl in two of the uridines, with all other ribonucleotides remaining unmodified. They observed that the modifications alter the dissociation pathways for UVPD and a‐EPD but give extensive backbone cleavage such as a/w, b/x, c/y, and d/z, which could be used to characterise and localise the modifications for oligoribonucleotides. A high number of base‐loss product ions and c/y ions are observed when HCD (off‐resonance) is used, this being independent of the modification present in the sequence. However, the presence of LNA or 2′‐*O*‐methoxyphosphorothioate in the sequence hinders dissociation when using UVPD compared to a‐EPD and HCD (off‐resonance), which are not significantly affected by the modifications. As a consequence, a‐EPD and HCD (off‐resonance) can be used for localisation of those modifications with complete sequence coverage. In the case of LNA, they explain this suppression due to the stabilisation of the *N*‐glycosidic bond and the lack of a transferable 2′‐proton. When PS is present in the sequence, it does not cause a significant suppression for a‐EPD nor for UVPD, and UVPD has a better sequence coverage compared to a‐EPD or HCD (off‐resonance). Furthermore, UVPD at 213 and 193 nm gives similar extensive backbone fragmentations and sequence coverage and could be used for localising the modification in the oligonucleotide sequence. They conclude that UVPD and a‐EPD could help in the sequencing and localisation of modifications in RNA therapeutics as these techniques gave high sequence coverages and produced lower abundances of base‐loss products common to CID (both off‐ and on‐resonance) methods.

**FIGURE 13:**
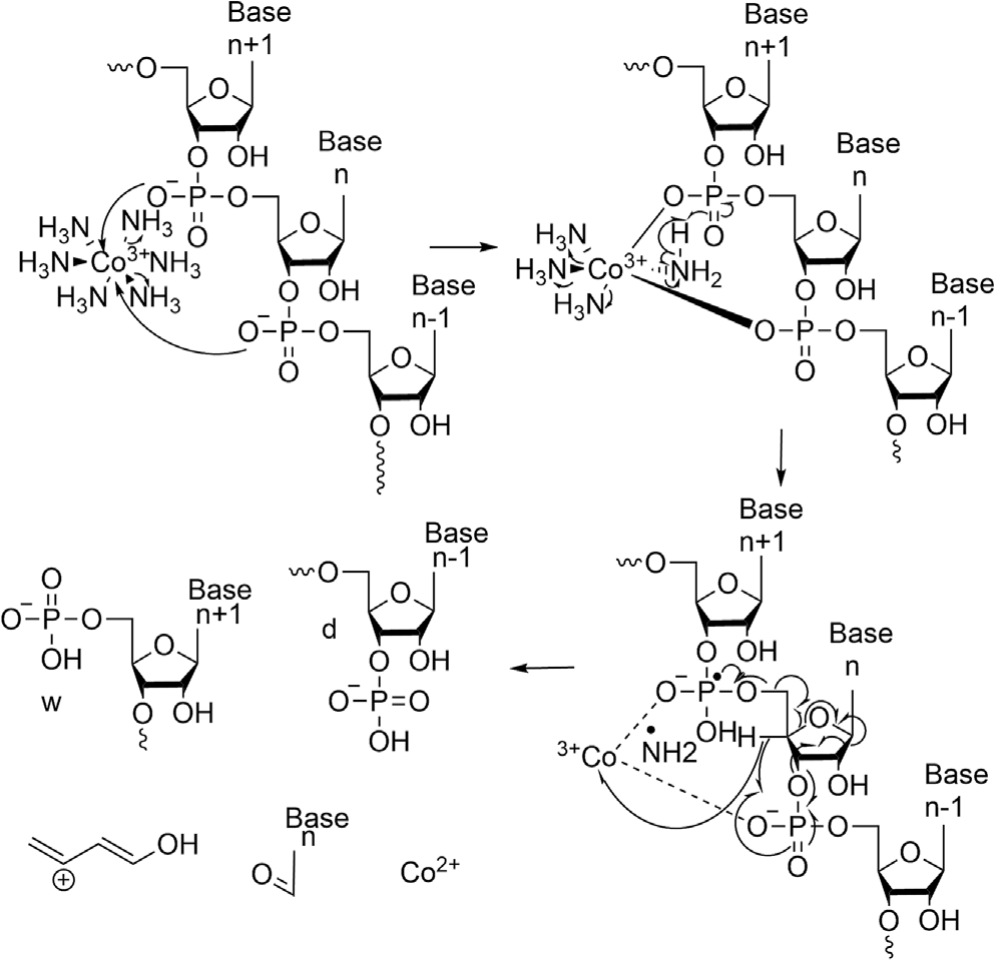
Proposed mechanism for the formation of the product ions d and w by RTD. Reproduced from Calderisi et al.^79^

Finally, new dissociation techniques have been used to characterise oligonucleotides. The first technique, called radical transfer dissociation (RTD), uses cobalt(III) hexamine ([Co^III^(NH_3_)_6_]^3+^) as reagent for the production of RNA radical ions.^79^ This enables ETD‐type experiments without the need for instrumentation equipped with this activation technique. The radical ions dissociate into d, w, c, and y product ions (with or without cobalt adducts) following supplemental collisional activation. The addition of Co adds more complexity through further salt adduction observed in the mass spectra, making manual processing more time consuming. To avoid this inconvenience, existing algorithms or software need to be adapted to automate the data analysis. The dissociation process, to form c and y product ions, follows the general process of RNA. A mechanism for the formation of d and w product ions has been proposed, which does not involve the nucleobases or the 2′‐OH groups, as shown in Figure 13, but is affected by the charge of (M + Co^III^(NH_3_)_6_ − *n*H)^(*n*−3)−^ ions. In the first step of the mechanism, [Co^III^(NH_3_)_6_]^3+^ is linked to two adjacent phosphodiester moieties, followed by loss of two ammonia molecules. Then, the abstraction of one H^•^, from a coordinated NH_3_, by a phosphodiester moiety liberates three other ammonia molecules. Consequently, a phosphoranyl radical is obtained which reacts by elimination of a buta‐1,3‐dien‐1‐ol cation and a nucleobase aldehyde. At the same time, the Co^3+^ is reduced to Co^2+^, which binds electrostatically to d or w product ions.

Finally, RTD was applied on modified RNA, such as 5‐hydroxymethylcytidine and 5‐formylcytidine, to study their stability and the energy necessary to fragment them compared to CID.^79^ The authors conclude that due to the binding of [Co^III^(NH_3_)_6_]^3+^ to the molecule, it affects its properties such as increasing the stability of the glycosidic bond or lowering the energy required for backbone cleavage. They suggest that this ‘new dissociation technique, RTD, allows for *de novo* sequence characterisation of modified RNA without the need for laborious sample preparation or specialised MS instrumentation’. It could be interesting to apply this technique to a wider range of modifications to RNA as well as to DNA and to compare the different sequence coverages to those of CID to determine its advantages and limitations.

The second new technique, ‘plasma’ EDD, which is an electron‐activated dissociation (EAD) method, was developed by Karasawa et al.^80^ for fast sequencing of oligonucleotides. Plasma EDD is performed in a neutral electron–nitrogen plasma stored in a magneto radio‐frequency ion trap using an electron beam kinetic energy of approximately 25–35 eV for 10–20 ms. This method can only be applied to highly charged ions, which produce spectra rich in product ions. Conversely, low‐charge states produced almost no product ions. The authors applied this technique first to unmodified DNA and RNA sequences, where plasma EDD produced fewer internal product ions than CID (off‐resonance). DNA sequence gave mainly a^•^ and w product ions and some c, d, x, and z^•^ ions. a‐B ion was negligibly weak compared to CID (off‐resonance), and a full sequence coverage was obtained using only a^•^ and w ions. On the other hand, RNA gave mainly d and w product ions and minor a^•^. The d and w ions have the same masses when the base elements in each product ion are the same, which can introduce false positives. Full sequence coverage was obtained by using d, w, and a^•^ product ions. When a sequence of DNA with full PS modification was analysed, a^•^ and w were dominant as for the previous unmodified DNA sequence. The presence of PS modification gave a low‐intensity charge‐reduced intermediate state [M − *n*H]^•(*n*−1)−^ and a high dissociation efficiency. However, when PS and PO were present in the DNA sequence, the cleavage at the PS portions was 100 times more intense than those at PO compared to CID (off‐resonance) where no significant dissociation efficiency was observed. In plasma EDD and CID, full sequence coverage was obtained. When LNA was present near the terminals, the dissociation was suppressed in plasma EDD at this modification position where only weak w product ions could be detected. In contrast, CID (off‐resonance) produced mainly b, c, f, x, and y product ions. The authors suspect that the dissociation mechanism of plasma EDD depends on the structure of the ribose and the linker group, which can be useful information for the radical‐induced reaction mechanism, but this was not studied in their publication. The authors also applied plasma EDD to impurities where the modifications could be localised and where in the sequence PS to PO occurs. Finally, they applied plasma EDD to a 40‐mer RNA where full sequence coverage was obtained (comparison to CID (off‐resonance) was not performed). They suggest that plasma EDD can provide full sequence coverage for approximately 50‐mers, but it was only applied to one 40‐mer RNA sequence without modification in their study. The authors detected cross‐ring cleavage in the ribose for the sample (dT)_15_, which could provide a useful tool for ribose modification localisation for a number of sequences. This new dissociation technique still needs to be tested with other type of modifications, for example, ribose modified at 2′‐position, to evaluate the broader utility of the approach for characterising oligonucleotides. Furthermore, plasma EDD induced secondary EDD products from charge‐reduced intermediate states, which produced product ions differing by the mass of a single hydrogen radical such as [a − H]^4−^ rather than a^•4−^, which can produce complex spectra. The authors found an empirical way to estimate the H^•^ mass shift using a model and obtained the formula $k=\left(\left|z^{*}\right|-\left|z\right|\right)$, where $z^{*}=\left(Z+1\right)\times m/M$ with *M* and *Z* (negative value) representing the precursor mass and charge respectively, *m* and *z* the experimental product ion mass and charge respectively, and z* the charge state of the product ion produced by the first EDD.

## 5 APPLICATION OF IMS TO THE CHARACTERISATION OF OLIGONUCLEOTIDES

IMS has been used together with CID to characterise oligonucleotides. For example, Ickert et al. studied the general behaviour of oligonucleotides by CID (on‐resonance) and IMS, in negative ESI mode, depending on their charge states.^81^ The authors observed that at a low charge state, a small collision cross section (CCS) is obtained, which means that the molecule is folded. Furthermore, high energy is necessary to fragment this ion. When the charge density increases, Coulombic repulsion increases, destabilising the molecule. As a consequence, the molecule starts to unfold, which is observed by an increase of the CCS. Additionally, the energy necessary to fragment the molecule decreased due to the destabilisation. The CCS increases only at a transition zone, which is situated between two linear zones: one at low and the other one at high charge levels. This signifies that the molecule begins as folded/compact and finishes as an unfolded/linear structure to become more stabilised and to reduce the Coulombic repulsion, which has a high influence on the dissociation behaviour of the molecule. This observation does not depend on the sequence and size but mainly on the charge density of the ion. Furthermore, at a low charge state, the loss of neutral base was obtained, but at a higher charge state the Coulombic repulsions increases. Consequently, the loss of charged base was observed to decrease the charges on the remaining ion. This transition is observed when around 50% of the molecule is charged, or in other words, when every other phosphate carries a negative charge. Those observations were also studied by Omuro et al.^82^ and more specifically the effect of the oligonucleotide sequence and length differences on the CCS values by traveling wave ion mobility spectrometry (TWIMS). They observed that when the charge states reached approximately one charge per 3.5–4 bases such as −4 to −5 charge states for 16‐mers, an inflection point was observed, which indicates that the oligonucleotides adopt two conformations in the gas phase, where the conformational change seems to occur around the inflection point. The authors concluded that the conformational change is related to negative charge repulsion in the gas phase where a folded structure is obtained for lower charge states compared to high charge states that are unfolded. When a modification is present in the oligonucleotide sequence, such as PS or LNA, the CCS values at lower charge states are proportional to the molecular weight and independent of the modification. However, in the intermediate charge state and above, the CCS values correlate to the modification. The authors observed that by increasing the number of PS in the oligonucleotide, the CCS width range increased by 70–110% for high charge states compared to low charge states, where the increase was 10–30% (the authors investigated −2 to −9 ions). The broadening of the peaks was a reflection of the increasing number of unresolved diastereomer species. Insufficient mobility resolution was available in this linear TWIMS‐based study to achieve separation of the 20‐mer oligonucleotide diastereomers. However, newer cyclic TWIMS technology may enable successful separation of the different species.^83^

Another study of the dependence of the CCS value on the charge state using negative ESI was reported by Arcella et al.^84^ The authors observed that the ‘bias voltage (using TWIMS), or the buffer composition’ do not have any influence on the CCS value and concluded that oligonucleotides lose the memory of the solution‐phase conformation when it enters the gas phase. As a consequence, it is not a metastable conformation, and the structure is different in solution and in the gas phase. Furthermore, they observed that proton transfer is common, even at low‐charge states, which can involve, mostly, phosphate‐to‐phosphate but also, less commonly, phosphate‐to‐nucleobase transfer. This observation of proton transfer phosphate‐to‐nucleobase could confirm different mechanisms such as of Wang et al.^49^ or Stucki et al.^53^ where the proton is transferred from phosphate to a nucleobase.

IMS can be also used to separate diastereomers as demonstrated by Demelenne et al.^85^ where the authors used drift tube IMS (DTIMS) and multiplexed IMS to separate diastereomers of 5‐mer oligonucleotides containing 0, 1, 2 or 3 PS linkages as sequence 5′‐TCGTG‐3′, in negative ESI. When oligonucleotides were analysed by DTIMS (single pulse) one peak for compound 0 (oligonucleotide without PS linkages) was measured, then two for both compound 1 (oligonucleotide with one PS linkage, two diastereomers) and compound 2 (oligonucleotide with two PS linkages, four diastereomers). For compound 3 (oligonucleotide with three PS linkages, eight diastereomers), there was only evidence of partial separation, and the eight expected diastereomers could not resolved. Multiplexed IMS allows ions to be released into the drift tube in short pulses according to a pre‐encoded sequence, instead of all at once, and therefore multiple ion packets are present at the same time in the drift tube. This results in increased duty cycle and therefore improved signal to noise. By using it, one single peak was observed for compound 0, two peaks were observed for compound 1, four peaks were obtained for compound 2 with one of them being much lower intensity and eight peaks were observed, but with different intensities, for compound 3. Separation of diastereomers can be important to understand if there is any difference of dissociation pathways between Sp and Rp configurations, but also as fully Sp‐configured oligonucleotides are resistant to nuclease degradation but have poor binding affinity compared to fully Rp‐configured oligonucleotides, which are less resistant to nuclease degradation but bind more strongly to the RNA target. This technique needs to be evaluated for longer oligonucleotide sequences with different types and numbers of modifications to understand its limits, for example, how many PS can be present in the sequence before separation by multiplexed DTIMS is not possible.

The cited studies show that, at the present time, it is challenging to separate intact oligonucleotide diastereomers using commercial instrumentation. It remains to be seen whether IMS analysis of product ions derived from different oligonucleotide diastereomers can be used to determine the stereochemistry of the precursor molecule, as has been demonstrated for peptides^86^ and oligosaccharides.^87^

### 5.1 Internal rearrangement by CID and IMS

After the first dissociation of the precursor ion, different product ions are obtained, which can fragment again if enough energy is present. If the product ion does not have the initial terminals of the molecule, it is called an internal product ion. This can correspond to the loss of a functional group present at each terminal, but it can also be the loss of different nucleotides. Depending on the chemistry of the oligonucleotide, internal rearrangements could be possible where two different parts of the sequence react to produce a product ion with a different sequence from the initial molecule. Those internal product ions and rearrangements have not yet been studied and understood which could give complementary information to McLuckey series ions. This would lead to a more complete interpretation of spectra and more confidence in structure assignment notably for unknown sequences such as impurities and degradants in pharmaceuticals.

**FIGURE 14:**
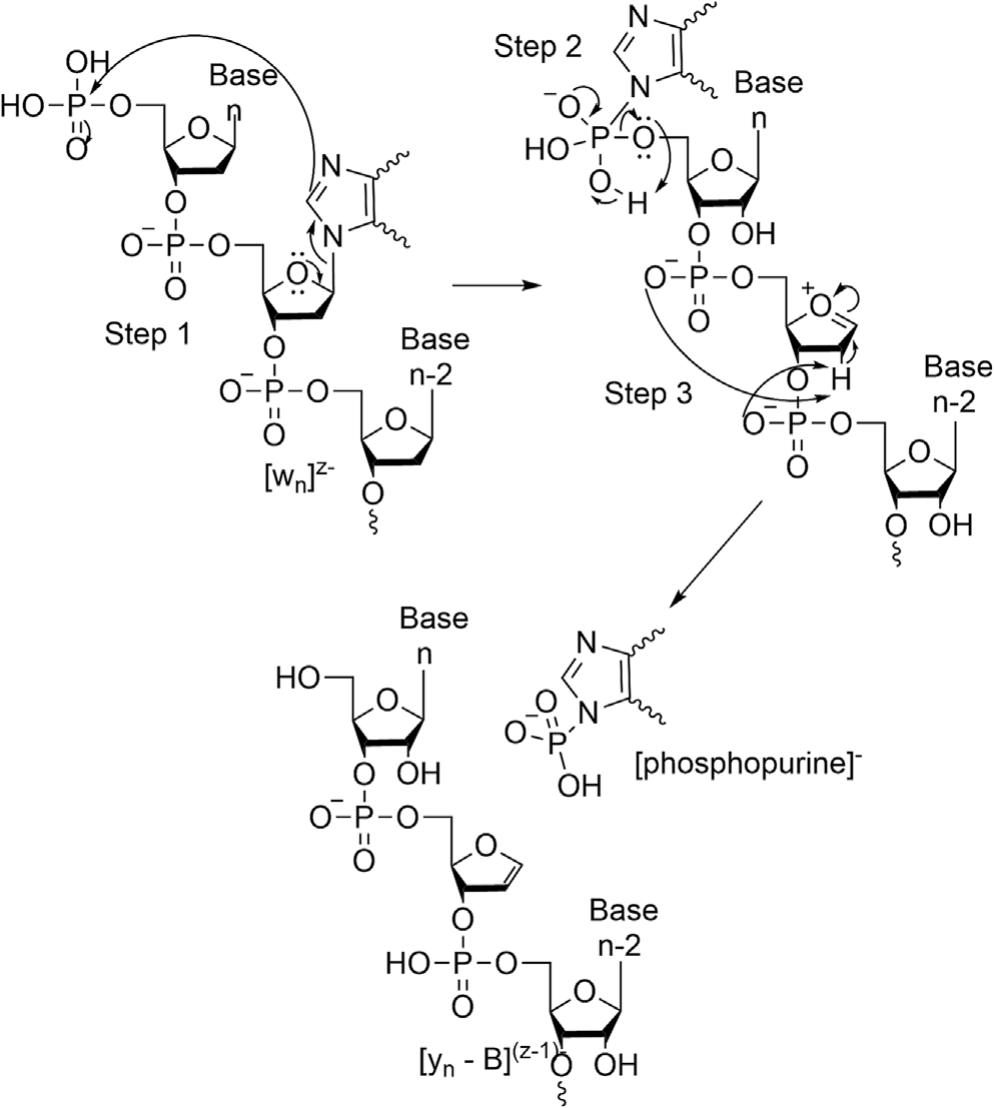
Proposed mechanism for the generation of rearranged [phosphopurine]^−^ and y‐B ions. Adapted from Harper et al.^88^

An internal rearrangement has been observed by Harper et al.^88^ where they used CID (both off‐ and on‐resonance) and IMS, in negative ESI mode, on isolated w product ions but also intact 5′‐phosphorylated DNA, corresponding to a w product ion, to obtain rearranged product ions. They concluded that a purine base can attack the free 5′‐phosphate group, as present in w product ion or 5′‐phosphorylated for DNA, to generate a rearranged phosphopurine and complementary y‐B product ion. This is supported due to the observation of phosphoadenine and phosphoguanine at *m*/*z* 214 and *m*/*z* 230, respectively, but also the complementary product ions y‐A and y‐G, respectively, in the CID (both off‐ and on‐resonance) mass spectrum of w ions as well as for 5′‐phosphorylated oligonucleotides. Furthermore, the formation of phosphoadenine is more important than phosphoguanine due to [A]^−^, which is more stable than [G]^−^. Those observations were also supported by theoretical calculations using density functional theory at the B3LYP/6‐31(d) level of theory and basis set,^88^, ^89^ which suggest that the formation of charged PO_3_^−^ and a corresponding neutral purine have lower energy dissociation pathways for [phosphopurines]^−^ than for the formation of neutral HPO_3_ and purine ions. The only product ion that cannot produce a phosphopurine is w_1_. This was hypothesised by the authors to be due to structural rigidity, which may restrict rearrangement reactions. However, when a w_2_ product ion, containing one A and one G, is analysed, both phosphopurines are obtained. They explained that it can be due to the existence of other lower energy dissociation pathways for [w_1_]^−^, such as those resulting from charge localisation. Furthermore, they explained that charge localisation to the 5′‐terminal phosphate group could increase the proton affinity of the neighbouring A and direct dissociation through loss of A, rather than the generation of [phosphoadenine]^−^ for w_1_. Analysing the phosphopurine by IMS showed only one peak, which suggests the presence of a single type, but multiple isomers or structures can be present and require higher mobility resolving power to separate them. This could be possible by using cIM,^83^ which increases the resolution after each pass around the mobility device.They suggest that phosphopurines can be generated from a common dissociation pathway such as [w_x_]^n–^ → [y_x_ − B]^(n − 1)−^ rather than from independent losses, which means [w_x_]^*n*−^ → [y_x_]^(*n* − 1)–^ → [y_x_ − B]^(*n* − 1)−^. When several G or A are present in the molecule, it is not possible to determine which one is lost, and as a consequence, the phosphoguanine and phosphoadenine could be a mixture of the different purines lost. The authors observed that the charge state, up to three, or length of the precursor ion did not affect the generation of phosphopurine. It is necessary to evaluate the consequence of a higher charge state to evaluate if the same observations could be obtained. Finally, no phosphorpyrimidine product ions were observed, and the authors suggest that ‘preferential generation of [phosphopurine]^−^ over [phosphopyrimidine]^−^ suggests that the bicyclic nitrogenous ring structure of purines might be essential for formation of intermediate and/or final product ions and their stability’. The proposed mechanism for the generation of phosphopurine and the complementary y‐B product ion is shown in Figure 14. Here, they proposed that first a pair of electrons from the ether oxygen (of the deoxyribose sugar) initiates cleavage of the purine base from the sugar backbone, followed by subsequent nucleophilic attack of the 5′‐phosphate group to form a phosphoamide tetrahedral transition species (Figure 14, Step 1). Then, the phosphoamide tetrahedral transition species can rearrange to generate two [phosphopurine]^−^ species at *m*/*z* 214 (phosphoadenine) and *m*/*z* 230 (phosphoguanine) (Figure 14, Step 2) and their complementary y‐B type ions. Finally, the neutralisation of the oxonium ion may be achieved by β‐elimination of a proton from the deoxyribose sugar by one of the adjacent phosphate groups (Figure 14, Step 3).

The authors concluded that additional analysis can be achieved on the other type of intact product ion, for example, a, b, c, d, x, y, and z type ions to obtain similar observations, but proof of that has yet to be reported in the literature. Furthermore, it could be interesting to evaluate this observation on other types of oligonucleotides such as RNA, with or without modifications, as here the authors used only unmodified DNA with natural bases.

## 6 CONCLUSION

The increased interest in oligonucleotides has driven demand for new tools and approaches for their characterisation. In recent years, new observations and mechanisms have been mainly reported for modified oligonucleotides as well as on the third‐generation molecules such as methyl nucleobase isomers and LNA. These observations have been made possible through the use of different dissociation techniques, as well as the development of new activation approaches such as RTD and plasma EDD. The increased interest in IMS, alongside improvements in the instrumentation, has also enabled greater understanding of oligonucleotide fragmentation and promises to reveal further new knowledge in years to come. Moreover, it has been observed that depending on the oligonucleotide analysed, different activation techniques can be used in a complementary manner to CID, which is presently the most commonly used approach to fragment oligonucleotides. Based on the observations made by the studies discussed above, it is possible to recommend the preferred ion activation technique(s) for different chemistries of oligonucleotide (Table 2). Judicious choice of the activation technique gives the best chance of success in characterising a sequence with confidence. This selection process is underpinned by understanding the gas‐phase chemistry, which is why studies are important and needed. However, one limitation for many researchers is having access to the activation techniques as many of them are not commercially available.

**TABLE 2:** Summary of ion activation technique(s) of choice depending on the chemistry of the oligonucleotide

	Ion activation techniques of choice	Ref.
Sequence rich in T with PS in DNA	HCD (off‐resonance), with or without CID (on‐resonance)	Abdullah et al^74^
Sequence rich in C and modified	ETD, complementary to CID (off‐resonance)	Hari et al^76^
RNA sequences	a‐EPD and UVPD for localisation of modification in RNA	Santos et al^78^
LNA modification	a‐EPD and HCD (off‐resonance)	Santos et al^78^
Sequence with PS and for localisation of modifications	UVPD	Santos et al^78^
Modified and unmodified sequences	NETD and AI‐NETD, complementary to CID (off‐resonance)	Peters‐Clarke et al^75^

Finally, the understanding of the gas‐phase chemistry of oligonucleotides is incomplete, so further work, notably for internal product ions, is required to characterise oligonucleotides of different lengths and sequences, and bearing different modifications, faster and more confidently. Through enhanced understanding of gas‐phase ion chemistry and fragmentation, better known and unknown oligonucleotide characterisation will be achievable.

### PEER REVIEW

The peer review history for this article is available at https://www.webofscience.com/api/gateway/wos/peer-review/10.1002/rcm.9596.

## Data Availability

No data due to manuscript reviewing existing literature.
